# Iliac venous stenting provides long-term relief from chronic pelvic pain

**DOI:** 10.1016/j.jvsv.2024.101993

**Published:** 2024-10-12

**Authors:** Laurencia Villalba, Theresa Larkin

**Affiliations:** a Graduate School of Medicine, University of Wollongong, Wollongong, NSW, Australia; b Vascular Surgery Department, Illawarra Shoalhaven Local Health District, Wollongong, NSW, Australia; c Vascular Care Centre, Wollongong, NSW, Australia

**Keywords:** Chronic pelvic pain, Venous stents, Intravascular ultrasound, Pregnancy, Pelvic venous disorders

## Abstract

**Objective:**

Iliac venous obstruction has been reported as a cause of chronic pelvic pain (CPP), however, there is a paucity of data in the literature reporting outcomes of venous stenting in this population. This study reports on a group of women with CPP and evidence of iliac venous obstruction: (1) the long-term impact of iliac vein stenting on pain scores; (2) the associations of age, stenosis severity, and concurrent presence of ovarian vein reflux (OVR) on pain; and (3) the effect of pregnancy after stenting.

**Methods:**

We conducted a retrospective analysis of prospectively collected data of women with chronic pelvic pain who subsequently underwent iliac vein stenting. Data analyzed included demographics, venous measures (iliac and ovarian veins), visual analog scales, and pregnancy after stenting.

**Results:**

A total of 113 female patients who had a history of chronic pelvic pain and underwent iliac venous stenting were included in analyses. The mean age at the time of stenting was 46.5 ± 15.7 years (range, 17-88 years). The baseline left common iliac vein diameter on duplex was 0.43 ± 0.18 cm and left common iliac vein area stenosis on intravascular ultrasound was 77.4 ± 9.4%. The baseline pain severity was correlated with younger age, degree of stenosis and presence of OVR. At a median follow-up of 5 years after stenting, 98% had improved pain scores and 73% had complete resolution of their pain despite the presence of residual OVR. Pregnancy after stenting did not result in the recurrence of pain and there were no stent-related complications with pregnancy.

**Conclusions:**

Iliac venous stenting provides long-term relief from CPP even with residual OVR and poststent pregnancy. With 73% of women having full pain resolution, and the rest having a mean residual pain score of <3, this study supports venous stenting for the treatment of CPP of venous origin, especially in young women.

**Article Highlights**

• **Type of Research:** Single-center observational study with retrospective analysis of prospectively collected data
• **Key Findings:** Of 113 women with chronic pelvic pain treated with venous stenting, 98% improved and 73% had complete resolution of pain over a median follow-up of 5 years despite residual ovarian incompetence and pregnancies after stenting. Baseline pain severity was correlated with younger age and degree of stenosis.
• **Take Home Message**: Iliac venous stenting provides long-term relief from chronic pelvic pain of venous origin.

Chronic pelvic pain (CPP) is prevalent in 6% to 27%^1^ of women of reproductive age. However, ≤40% of these women do not have a clear diagnosis for their CPP after conventional evaluation.^2^ Most women with CPP are not assessed for pelvic venous disorders (PeVDs) as a cause of their pain,^3^ despite reports stating that this could be the main diagnosis in as many as 30% and a secondary diagnosis in another 10% to 15% of cases.^4^ CPP caused by PeVD is related to venous hypertension caused by ovarian vein reflux (OVR) and/or iliac venous obstruction (IVO), with reflux into the internal iliac vein^4^^,^^5^; however, most patients with CPP are not assessed to ascertain the presence of IVO. As a result, most of the literature on interventional management of PeVD causing CPP refers exclusively to ovarian vein embolization.^6^

Despite international guidelines suggesting that IVO can cause disabling pelvic pain and well-selected patients can benefit from venous stenting,^5^ there is a paucity of data in the literature reporting outcomes of venous stenting in this population. A recent review^7^ suggested that venous stenting should be considered in females with PeVD and IVO. Furthermore, there is uncertainty on the best strategy for when IVO and OVR are both present with a recent commentary highlighting that more research on the outcomes of iliac vein stenting in is needed.^8^ We could only find four previous studies that specifically reported on the outcomes of iliac venous stenting in a population with CPP, published between 2015 and 2021.^9^, ^10^, ^11^, ^12^ The median follow-up of these was <12 months. All four studies reported vein diameter and/or percent stenosis, with three using intravascular ultrasound (IVUS) examination and reporting on pain scores. There was a mix across studies in terms of whether patients did or did not undergo ovarian vein coiling. None of these studies examined the associations between vein measurements, age, and pain scores, nor the effect of pregnancy after stenting on pain scores.

The aims of the current study were to determine in a cohort of women with CPP and evidence of IVO (1) the long-term impact of iliac vein stenting on pain scores, (2) the associations of age, left common iliac vein (LCIV) stenosis severity, and concurrent presence of OVR on pain measures (pain severity at baseline and ≤5 years after stenting, change in pain severity over time including full resolution), and (3) the effect of pregnancy after stenting on pain scores.

## Methods

### Ethics approval and study design

This study gained ethics approval from the University of Wollongong Human Research Ethics Committee (ethics approval number 2017/409). We conducted a retrospective analysis of prospectively collected data of women who presented to the Vascular Care Centre, Wollongong, Australia, from 2016 to 2023 who underwent iliac vein stenting and in their preoperative questionnaire had mentioned pelvic pain.

### Preoperative assessment

Patients referred for lower limb venous symptoms or pelvic pain presumed to be of venous origin answered a questionnaire including a visual analogue scale for pelvic pain before the first consultation with a vascular surgeon. Villalta scores were assessed for patients with post-thrombotic syndrome (PTS). Patients whose chief complaint was pelvic pain were assessed using a dedicated pelvic pain questionnaire, a pain diary for 6 weeks and a review by a specialist gynecologist to rule out any other potential diagnosis.

All patients presumed to have a pelvic source of their pelvic and/or lower limb symptoms underwent a formal transabdominal duplex ultrasound (TAU) examination of the abdominopelvic veins to assess OVR and obstructive lesions of the left renal vein and the iliocaval veins, following a dedicated protocol that has been previously published.^13^ In brief, TAU examination was performed using B-mode with color flow Doppler (Toshiba Aplio 300 platinum series; Canon Medical Systems, Otawara, Japan) with a 1.9- to 6.0-MHz convex array transducer in the transverse view. All ultrasound scans were performed in the morning, with the patient fasted but hydrated (patients are told to drink 2-3 L of water the day before and 2-3 glasses of water in the morning the day of the TAU examination). The patients were placed supine in the reverse Trendelenburg position (45°) with their arms at their side or comfortably on their chest. Once a target lesion was identified, it was measured at rest and during a Valsalva maneuver to assess for dynamic compression vs true stenosis. Intraluminal details like thickening, webs, or scarring were also recorded. The minimum diameter at the target lesion and the diameters of the other veins of the iliocaval system were measured. The percentage stenosis was calculated both relative to a normal segment of the ipsilateral or contralateral CIV and using the literature-derived CIV diameter of 16 mm. This is in line with a recent study that reported it is essential to include normalization of the iliac veins to an internal control or a standard diameter measure.^14^ We use the literature-derived reference diameter to minimize the potential for overestimating the stenosis by using a denominator that could have been abnormally dilated by the stenosis or compensatory flow. Loss of phasicity and evidence of collateral pathways was also reported when present. We do not report velocities.

From our previous validation study, we found that a TAUS-measured vein diameter of 8 mm equated to an IVUS cross-sectional area of 53% stenosis, with the TAUS minimum diameter and IVUS minimum area measures significantly correlated, so we defined a significant obstructive lesion on the common iliac vein as a diameter of <8 mm fixed stenosis; significant renal vein compression of the left renal vein was indicated by a 5:1 diameter and velocity ratio,^15^ and ovarian reflux was defined as any retrograde flow.^16^

### Patient selection and intervention details

Patients whose duplex ultrasound examination indicated CIV stenosis and those thought to have clinically relevant IVO with disabling symptoms even in the absence of venous pathology on duplex ultrasound examination were offered invasive imaging with venography or IVUS examination with intention to treat, after a trial of conservative management of ≥6 months. Clinically relevant IVO, caused by PTS or nonthrombotic iliac vein lesions (NIVLs) was defined as a Clinical, Etiologic, Anatomic and Pathophysiologic (CEAP) class 3 to 6 and/or a Venous Clinical Severity Score pain score of ≥2, or disabling pelvic pain, deep dyspareunia, or postcoital pain when other causes had been ruled out by a specialist gynecologist.^5^ Data analyzed in the current study were of all patients who mentioned pelvic pain in their preoperative assessment questionnaire regardless of whether it was their chief complaint or not.

IVUS examination was only available to insured patients up until 2018, but after this, all patients had access to IVUS examination. Our IVUS protocol has also been previously published,^17^ but in summary, multiview venography of the bilateral limbs is used as a roadmap followed by the Visions PV .035 digital IVUS catheter (Volcano/Phillips). We perform the procedures under general anesthetic and prehydrate the patients with 1000-mL bolus of intravenous normal saline. A slow pullback on a recording mode allows for assessment of each vein segment during a respiratory cycle, paying close attention to intraluminal details. Once a lesion is identified, measurements are performed under the Valsalva maneuver.

Patients with clinically relevant IVO and a significant stenosis, defined as >50% on multiview venography and/or IVUS examination, received balloon angioplasty and dedicated venous stents as per international guidelines.^5^ after stenting, IVUS examination was used again to assess stent expansion, lumen, and apposition.

### Follow-up

Clinical follow-up and duplex ultrasound examination were performed at 1, 3, 6, 12, 18, and 24 months, and yearly thereafter. Pelvic pain severity was assessed using visual analogue scales, and Villalta scores were assessed for the patients with PTS at each postoperative visit.

### Statistical analyses

Mean values and standard deviations were used to report all continuous variables. Baseline pain was compared between category of duration of CPP with unpaired *t* tests, and between categories of ovarian vein competence, reflux, previous coiling via one-way analysis of variance (ANOVA) with post hoc analyses with Bonferroni correction. Repeated measures ANOVA was used to determine the change in pain scores across time before and after stenting. Difference in pain scores across time between categories of venous pathology (LCIV obstruction only; LCIV obstruction + left OVR; LCIV obstruction + left OVR + left renal vein compression) was assessed via repeated measures ANOVA with between group analyses. Pearson's correlations were used to assess relations between age, LCIV and left ovarian vein diameters, baseline pain, latest pain score, and change in pain score. We performed χ^2^ tests to determine associations between ovarian vein category with renal vein compression and with pain resolution. Unpaired *t* tests were used to compare whether those with vs without full resolution of the pain differed in age at stenting or their vein diameters. The impact of poststent pregnancy on pain measures was assessed using χ^2^ tests for proportion achieving full pain resolution and using unpaired *t* tests for prestent and latest pain scores, and pain score change between these time points.

## Results

### Patient characteristics and pain scores

Data from a total of 113 female patients who had a history of CPP and underwent iliac venous stenting were included in analyses. The mean age at the time of stenting was 46.5 ± 15.7 years (range, 17-88 years). A total of 83 patients (73.5%) had signs of chronic venous disease, including three who were CEAP 5 and 10 patients (12.4 %) with PTS. Most patients (86.7%) also had leg pain, 58.4% had back pain, and 46% experienced dyspareunia. None of the patients had hematuria or flank pain. There were 13 patients who had previous ovarian coiling.

The mean pelvic pain score before stenting was 6.7 ± 2.3 (range, 1-10; median, 7). More than one-third of patients (35.4%) had been prescribed pain medication specifically for their CPP. The mean duration for which CPP had been experienced was 6.9 ± 6.4 years (range, 1-25 years). Baseline pain score was not significantly different for those who had pain for ≤5 years vs for >5 years (*P* = .535).

### Preoperative duplex data

On preoperative duplex ultrasound examination, 96.3% had evidence of a significant obstructive lesion on the LCIV. This included 35.5% who had bilateral common iliac vein lesions. The mean percent stenosis of the LCIV as per duplex was 66% ± 15% (range, 15%-92%). Vein diameters are presented in Table I.

**Table I:** Pelvic vein diameters for the cohort (n = 113) as measured via duplex ultrasound

Vein measure	Mean ± standard deviation, cm	Range, cm
LCIV smallest diameter	0.43 ± 0.18	0.11-1.1
LCIV largest diameter	1.3 ± 0.2	0.80-1.9
RCIV smallest diameter	0.9 ± 0.3	0.28-1.7
RCIV largest diameter	1.1 ± 0.3	0.40-1.9
LEIV smallest diameter	1.1 ± 0.3	0.2-1.7
LEIV largest diameter	1.1 ± 0.2	0.6-1.7
REIV smallest diameter	1.1 ± 0.2	0.7-1.6
REIV largest diameter	1.2 ± 0.3	0.6-1.7

*LCIV,* Left common iliac vein; *LEIV,* left external iliac vein; *RCIV,* right common iliac vein; *REIV,* right external iliac vein.

At baseline, 95 patients also had information on their left ovarian vein and 86 patients had measures of their left renal vein. In terms of the left ovarian vein, there were 42 (44%) who had antegrade flow, 13 (14%) who had previous coiling, and 40 (42%) who had retrograde flow. The mean diameter of left ovarian vein for all patients at baseline was 4.3 ± 1.8 mm (range, 2.1-11.2 mm). This included for those with a refluxing ovarian vein, a mean diameter of 5.4 ± 1.9 mm (range, 2.4-12.0 mm) which was significantly wider (*P* < .001) than for those with a normal left ovarian vein, of mean diameter 3.3 ± 0.71 mm (range, 2.1-5.0 mm). The majority (72%) had a normal renal vein and 28% had renal vein compression on duplex ultrasound examination.

Taken together, 19% of the cohort had left OVR and left renal vein compression, in addition to LCIV stenosis. There was a significant association between OVR (coiled and not coiled) and renal vein compression, χ^2^(2) = 11.372; *P* = .003. More than one-half of the patients (57%) who had a coiled left ovarian vein had left renal vein compression, and 44% of those with a refluxing left ovarian vein and only 11% of those with an antegrade left ovarian vein had left renal vein compression.

### Intervention details

All patients had evidence of significant pelvic reservoirs on venography with retrograde flow from the internal iliac vein and stagnation of contrast within the pelvic collaterals. Forty patients had IVUS measurements available, and all had >60% stenosis recorded. The minimum area and percentage of area reduction for the patients for whom there were IVUS measurements recorded were 50.0 ± 23.1 mm^2^ (range, 0-111.3 mm^2^) and 77.4 ± 9.4% (range, 60.7%-100%), respectively. The patients that had venography only had >70% stenosis recorded.

All patients received sinus-venous stents (Optimed Medizinische Instrumente GmbH, Ettlingen, Germany). The majority were 16 mm (n = 92 [81%]), and the remainder were 14 mm (n = 14 [12%]) or 18 mm (n = 7 [6%]). Stent length ranged from 60 to 230 mm with a median of 120 mm, and the majority (n = 95 [84%]) ≥100 mm, anchored in the external iliac vein. There were 34 (30%) bilateral stents.

### Technical results

There were no stent fractures or migration. For the post-thrombotic patients, two patients developed in stent thrombosis, one related to the coronavirus disease vaccine and the other owing to a technical error missing a distal lesion. For the NIVL group one patient developed subocclusive thrombosis due to lack of compliance with antithrombotic medication. All three underwent successful reintervention with pharmacomechanical thrombectomy. For the 10 patients with PTS, primary patency was 90% at 12 months and 80% at 5 years, and secondary patency was 100%. For the 103 patients with NIVL, the patency rates were 99% at 12 months, with an assisted primary patency of 100% at 5 years. Residual pelvic reservoirs were not treated in any of the patients and no patient received ovarian vein embolization.

### Pelvic pain scores after stenting

The latest pain score for all patients (median, 60 months; range, 3-72 months) was 1.3 ± 1.7 (range, 0-8; median, 0), and the mean change in pain score from baseline to latest follow-up was −5.5 ± 2.4 (range, 0-10). The majority (n = 82 [73%]) had complete resolution of their pain, achieved at a median of 6 months after stenting. For those who did not have complete pain resolution, their latest pain score was 2.8 ± 1.9, which was significantly lower than their pretreatment pain score of 7.5 ± 2.0 (*P* = .045). The only significant difference between those who did vs did not have complete pain resolution was that those who did not have complete pain resolution had a significantly higher baseline pain score than those who did (7.5 ± 2.0 vs 6.3 ± 2.3; *P* = .019). There was no difference between these two subgroups for any of age at stent, diameter, or percent stenosis of the LCIV before stenting, diameter of the left ovarian vein before stenting, or duration of pain.

**Fig 1:**
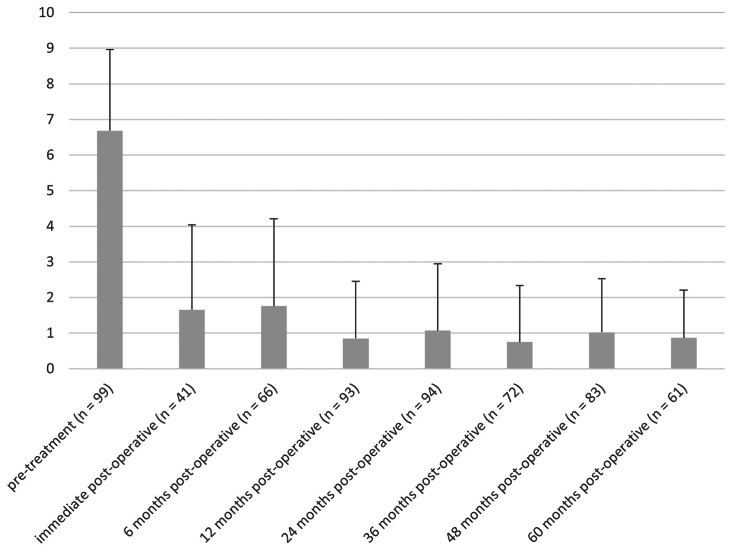
Visual analog scale pain scores before treatment and up to 60 months postoperative. Data are mean with standard deviation shown as error bars; n value is the number of patients with data at each time point.

Pelvic pain scores at baseline and to a follow-up of 5 years are presented in Fig 1. Repeated measures analysis for those patients who had pain scores recorded at each of baseline, and 6, 12, 24, 36, 48, and 60 months after treatment (n = 48) showed a significant effect of treatment on pain score, F(5,43) = 80.281; *P* < .0001. The baseline pain score was significantly higher than all pain scores after treatment (*P* < .0001 for all; Bonferroni post hoc analyses).

### Associations between age and pain measures

Age was significantly inversely correlated with baseline pain (R = −0.307; *P* = .002) and with latest pain score (R = −0.218; *P* = .034), but not with the change in pain score after stenting (R = −0.116; *P* = .252). There was no difference in age at stent between those who did vs did not have full pain resolution (*P* = .167). Age was significantly, weakly correlated with the baseline smallest diameter of the LCIV (R = 0.267; *P* = .006) and negatively, moderately correlated with percent LCIV stenosis on duplex ultrasound (R = −0.451; *P* < .001), but not with baseline left ovarian vein diameter (R = 0.042; *P* = .718). This association was such that younger patients had a smaller diameter and higher percent stenosis of the LCIV.

There were 14 women who were aged <30 years at the time of stenting. These reported symptoms since their teenage years at a mean age of 15.1 ± 1.6 years (range, 13-18 years). Their baseline pain score was 8.1 ± 1.9 and smallest LCIV diameter was 0.33 ± 0.1 cm (range, 0.15-0.51 cm). Their pain score was significantly decreased at 12 months after stenting (2.2 ± 2.1; *P* < .001) and latest follow-up (2.5 ± 2.0; *P* < .001), which was ≥24 months for 12 of these 14 patients (median, 48 months). Five of these had complete pain resolution.

### Associations between LCIV stenosis severity, ovarian vein diameter and reflux, and pain measures

Baseline pain score was significantly correlated with baseline LCIV and left ovarian vein measures: inversely with the narrowest diameter of the LCIV and positively with the percent stenosis of the LCIV on duplex and the ovarian vein diameter before stenting (Table II). There was also a significant impact of left OVR on initial pain scores, F(2) = 5.194, *P* = .008. Baseline pain scores for those with left OVR were the highest (7.6 ± 2.1): significantly higher (*P* = .016) than those who had previous ovarian coiling (5.6 ± 2.4) and almost significantly higher (*P* = .050) than those with a competent left ovarian vein (6.4 ± 2.0). There was no difference in pain scores for previous ovarian coiling vs a competent left ovarian vein (*P* = .814, all Bonferroni post hoc analyses).

**Table II:** Correlations between pain scores and left common iliac vein (*LCIV*) and left ovarian vein measures

	Baseline vein measure		
	Smallest LCIV diameter	% Stenosis LCIV	Left ovarian vein diameter
Baseline pain score	R = −0.282 *P* = .006	R = 0.332 *P* = .001	R = 0.262 *P* = .031
Latest pain score after stenting (median 6 months)	R = 0.027 *P* = .788	R = 0.022 *P* = .825	R = 0.246 *P* = .036
Change in pain score from baseline to latest after stenting	R = −0.252 *P* = .017	R = 0.211 *P* = .046	R = 0.095 *P* = .451

The latest pain score was significantly correlated with ovarian vein diameter before treatment, whereas the change in pain score was significantly associated with LCIV percent stenosis and the smallest diameter of the LCIV, though all were weak correlations (Table II). Whether there was complete resolution of the CPP was not impacted by the smallest diameter of the LCIV or the left ovarian vein diameter before stenting (*P* = .110 and *P* = .913, respectively), nor whether the left ovarian vein was refluxing, coiled or competent at baseline, χ^2^ (2) = 0.109, *P* = .947.

**Fig 2:**
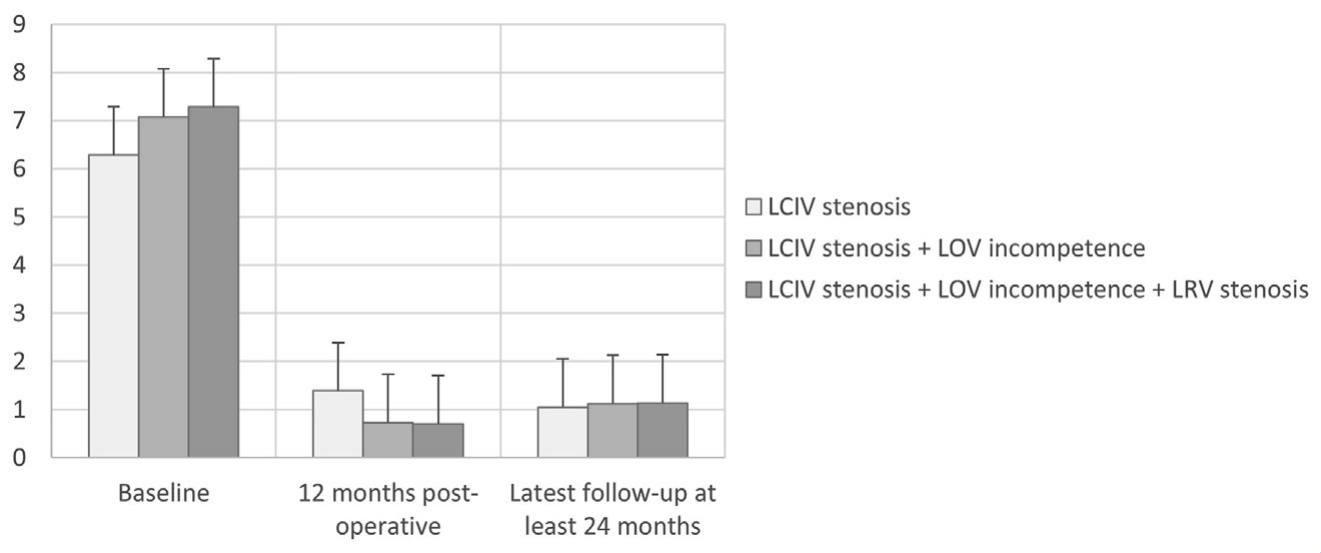
Visual analog scale pain scores before treatment and ≤12 months and ≥24 months postoperative. Data are mean with standard deviation shown as error bars. *LCIV*, left common iliac vein; *LOV*, left ovarian vein; *LRV*, left renal vein.

The significant decrease in pain scores over time, F_3,81_ = 313.129, *P* < .001; one-way ANOVA with repeated measures, was not different for those with LCIV stenosis only vs LCIV stenosis + left ovarian reflux vs LCIV stenosis + left OVR + left renal vein compression, F = 0.023, *P* = .978, between-groups analysis (Fig 2).

### Impact of stenting on left ovarian vein diameter and reflux, and renal vein compression

**Fig 3:**
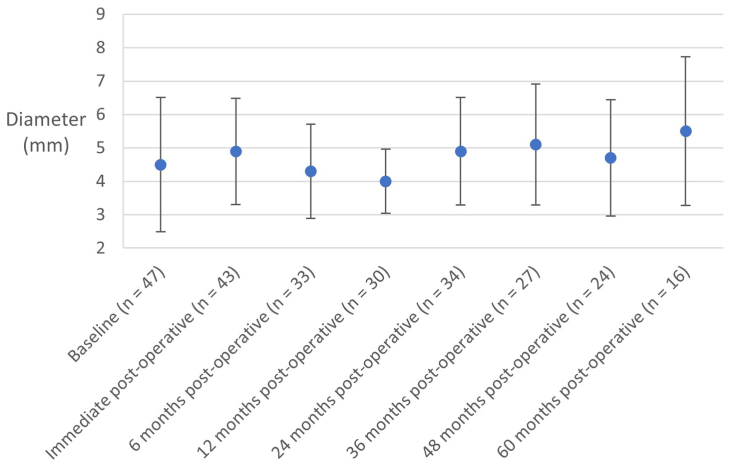
Left ovarian vein diameter at baseline and ≤60 months postoperative for all patients with measurements. Data are mean with standard deviation shown as error bars, and number of patients with data at each time point.

Left ovarian vein diameters for all patients are shown in Fig 3. The minimum mean left ovarian vein diameter at any of the time points was 4.0 ± 0.96 mm. The small sample size for repeated measures of the left ovarian vein diameter limited statistical analyses; however, it was clear that there was no impact of LCIV stenting on left ovarian vein diameter ≤60 months after stenting, regardless of whether they were refluxing preoperatively or not.

Among 60 patients who had measures of their left ovarian vein ≥24 months after stenting (median, 48 months), there was no impact of stenting on OVR: this had a prevalence of 56% at baseline and 57% at the latest measure. There was no association between OVR at the latest time point and complete pain resolution, χ^2^(1) = 1.663, *P* = .197: 74% of those with residual left OVR and 58% of those with a competent left ovarian vein had complete pain resolution. Among those with renal vein compression for whom there was sufficient data (n = 21), the renal vein compression reversed in 13 of these patients (62%) and 50% of 13 patients who had reversal of renal vein compression after iliocaval stenting also had a reversal of left OVR, compared with only 25% of the 8 patients whose baseline renal vein compression did not improve.

### Associations between pregnancy after stenting and pain measures

There were 71 patients <50 years of age at the time of stenting, and 12 patients had 16 successful pregnancies after stenting. Of these 12 patients, 8 had full pain resolution at a median of 6 months after stenting (median follow-up, 60 months). This proportion of women to have full pain resolution (67%) was not different to those who did not (65%) have a pregnancy after stenting, χ^2^ = 0.025, *P* = .874. There was also no difference between those who did vs did not have a pregnancy after stenting for pretreatment pain score (7.2 ± 1.5 vs 6.6 ± 2.4; *P* = .296), latest pain score (0.8 ± 1.4 vs 1.2 ± 1.8; *P* = .517) or change in pain score (5.8 ± 1.9 vs 6.0 ± 2.5[ *P* = .826). There was no recurrence of pain during or after pregnancy for the patients that had achieved full resolution and no worsening of pain in any of the other patients. There were no stent-related complications.

### Impact of stenting on leg pain

Leg pain was present in 99 patients preoperatively and improved in all of them. The patients with PTS had a median Villalta score of 17 points (range, 11-27) preoperatively and a median of 5 points (range, 0-10) postoperatively.

## Discussion

This series demonstrates the long-term effectiveness of iliac vein stenting for the treatment of CPP even in the presence of residual pelvic reservoirs and OVR. Although baseline pain severity was correlated with left ovarian vein diameter, the decrease in pain after stenting was not associated with baseline measures of the left ovarian vein or the presence of residual OVR. There was also no difference in prevalence of complete pain resolution for those with vs without residual OVR. Overall, our results agree with earlier studies^9^^,^^11^^,^^12^ of significant pain improvement and high percentage of complete pain resolution with iliac vein stenting despite the presence of residual pelvic reservoirs or OVR.

A study by Lakhanpal et al^11^ reported on 38 women with pelvic pain secondary to combined IVS and OVR, and despite having 76% achieved complete symptom resolution with iliac vein stenting alone, they suggested that, in some women, a relationship might exist between the presence of a pelvic reservoir and the persistence of symptoms and coiling should be considered if there is residual pain post stenting. In the current cohort, the latest mean pain score of those who did not achieve full pain resolution during the study period, was <3, which we did not consider high enough to trigger additional intervention.

The current study supports previous conclusions suggesting that when IVO and OVR are present concurrently, treating the IVO alone may be sufficient in most patients, especially with more severe stenosis. Santoshi et al^12^ reported an incidence of 80% of significant iliac vein stenosis (defined as >50%) in women with pelvic venous insufficiency and pelvic pain, when analyzing response to treatment in those with combined OVR and IVO they reported only 9 of 94 patients responded to OVE alone, and only after stenting the patients experienced significant benefit. Within our cohort, 13 patients had undergone previous OVE but due to unresolved CPP needed subsequent iliac venous stenting. Of note, we did not perform OVE after venous stenting in any of the patients in this series, regardless of the residual pelvic reservoir, over a period of 5 years. This is contrary to the recommendation from Santoshi et al^12^ and Lakhanpal et al,^11^ who recommend OVE if there is residual pain post stenting; however, they made the decision to proceed with OVE at 3 months while we have reported a median of 6 months to achieve full pain resolution in most patients with some taking 12 months to achieve that, so perhaps waiting 6 months before offering further intervention should be considered. Interestingly, 50% of 13 patients who had reversal of renal vein compression after iliocaval stenting also had a reversal of left OVR, compared with only 25% of the 8 patients whose baseline renal vein compression did not improve suggesting that the ovarian vein could have been acting as an outflow vessel. However, these findings are of small sample size, so need to be interpreted conservatively.

The baseline LCIV minimum diameter on duplex ultrasound examination was 0.43 ± 0.18 cm and the LCIV area stenosis on IVUS was 77.4 ± 9.4%, similar to that reported by others.^9^^,^^11^^,^^12^ All patients except two (98%) had improved pain scores, and 73% had complete resolution of their pain. Previous studies^11^^,^^12^ also reported that 74% and 76%, respectively, of their cohort had complete resolution of CPP after stenting for IVO, but these studies had <12 months of follow-up, whereas the current results demonstrate sustained benefit in pain reduction and/or elimination over a median follow-up of 60 months. This is particularly important considering that we found that the severity of the baseline pain was significantly associated with younger age.

Another finding in the current study, not previously reported, is that both prestent pain severity and reduction in pain severity after stenting were significantly correlated with baseline LCIV stenosis. This finding is in line with what the VIDIO (Venogram vs IVUS for Diagnosing IVO) trial investigators reported^18^ and has now helped our selection criteria for offering venous stenting to patients with disabling pelvic pain and >70% stenosis on imaging rather than >50%.

Finally, an important finding was that pregnancy after stenting, including multiple pregnancies, did not result in recurrence of pain and there were no stent-related complications with pregnancy.

The biggest limitation of the current study was that this was a retrospective analysis of data from a single center and included no formal quality-of-life assessment. However, visual analog scales for pain were used at every follow-up appointment and with a median follow-up of 5 years, we believe the results strongly support venous stenting for disabling CPP.

## Conclusions

Women suffering from disabling CPP should be worked up for the presence of venous outflow obstruction because iliac vein stenting provided significant long-term benefit with full resolution for the majority despite residual ovarian reflux and subsequent pregnancy. This strategy is particularly relevant for younger patients and with more severe left iliac vein stenosis, because these were both significantly associated with baseline pain severity.

## Author Contributions

Conception and design: LV

Analysis and interpretation: LV, TL

Data collection: LV

Writing the article: LV, TL

Critical revision of the article: LV, TL

Final approval of the article: LV, TL

Statistical analysis: TL

Obtained funding: Not applicable

Overall responsibility: LV

## Disclosures

None.
